# Gluten-Free Corn Cookies Incorporated With Stinging Nettle Leaf Flour: Effect on Physical Properties, Storage Stability, and Health Benefits

**DOI:** 10.1155/2024/8864560

**Published:** 2024-08-05

**Authors:** Mary Nkongho Tanyitiku, Prisca Bessem, Igor Casimir Njombissie Petcheu

**Affiliations:** ^1^ Women's Network for Biodiversity and Food Science, Yaounde, Cameroon; ^2^ Department of Veterinary Medicine Faculty of Agriculture and Veterinary Medicine University of Buea, Buea, Cameroon; ^3^ Global Mapping and Environmental Monitoring, Yaounde, Cameroon

**Keywords:** celiac disease, gluten-free cookies, glycaemic index food, nutrition, storage stability, *Urtica dioica* L. leaf flour

## Abstract

The consumption of gluten-free corn cookies is becoming very popular among nonceliac and celiac individuals. However, the absence of gluten and other nutrients in corn generally leads to cookies of lower quality in terms of nutritional value, texture, colour, and shelf life. To improve the quality characteristics of corn cookies, this study investigated the effect of incorporating an underutilised herb (*Urtica dioica* L. leaves) on its nutritional and physical properties. Stinging nettle leaf flour was incorporated at different levels (5%, 10%, 15%, and 20%) and compared with a control (100% corn cookies). The storage stability of the formulated corn cookies was also investigated at room and frozen (−18 ± 2°C) temperature. The incorporation of stinging nettle leaf flour increased (*p* < 0.05) the ash and protein content of corn cookies from 0.32% (control) to 2.56% (20% stinging nettle leaf flour incorporation) and 6.44% (control) to 21.52% (20% stinging nettle leaf flour incorporation), respectively. After in vitro starch digestion, the total phenolic content (TPC) and antioxidant activity (AA) also increased approximately 27 and seven times, respectively, and the estimated glycaemic index (GI) (eGI) decreased (*p* < 0.05) from 48.60% (control) to 33.18% (20% stinging nettle incorporated). Shelf life characteristics (water activity, peroxide value (PV), and microbial count) of formulated corn cookies were within acceptable limits for human consumption upon storage for 6 months. The findings indicated that stinging nettle leaves could serve as a potential food ingredient in gluten-free bakery products, particularly where low GI foods are desirable.

## 1. Introduction

Celiac disease affects more than 1% of people in most ethnic groups worldwide [1, 2]. It occurs when an ingested gluten diet causes swellings within the mucosal layer of the small intestines, leading to health conditions such as villous atrophy, diarrhoea, and chronic abdominal pain, among others [1, 3]. Currently, its only treatment is complete abstinence from gluten protein that is found naturally in foods such as wheat, rye, and barley [1, 2]. According to the US Food and Drug Administration, foods labelled “gluten-free” must contain less than 20 parts per million of gluten, an amount too small to cause harm to celiac patients [2, 4].

Corn is the most abundant gluten-free cereal due to its growing genetic potential and crop yield [1]. It is widely consumed as a staple or processed into various extruded and bakery products, including cookies [1]. In fact, cookies are a daily food for all age groups as they are delicious, are sold at reasonable prices, and are easy to transport to any occasion [5, 6]. However, unlike traditional wheat cookies, dough made from solely corn flour are limited in forming desirable gluten viscoelastic and cohesive networks [1–3]. This often led to major baking problems such as crumbling texture, poor colour, low specific volume, and shorter shelf life [1–3]. With this, food scientists are looking for new food ingredients that will provide adequate gluten-free nutrients, texture, and sensory properties at lower costs to consumers [3, 7].

Stinging nettle (*Urtica dioica* L.) is an abundant and nutritious perennial herb that was found to grow in temperate and tropical Asia, Europe, Northern America, and Africa [8–10]. In fact, they contain essential bioactive compounds with antioxidant, antimicrobial, antiulcer, and anti-inflammatory properties [9, 10]. Although stinging nettles are generally considered a key ingredient in traditional medicine or as tenacious weeds with stinging bites when touched on farmlands and/or footpaths [9, 11], some studies [10, 12, 13] have recently focused on the impact of adding nettle leaf flour in wheat bakery products. For example, an incorporation of nettle leaf tea in wheat bread was reported to cause a decrease in baking loss and bread volume, as well as an increase in the porosity index of the herbal breadcrumbs [13]. However, there is no data indicating its quality attributes, health benefits, and shelf life properties in gluten-free foods.

To contribute to the increasing demand and availability for healthy gluten-free foods, the purpose of this study was to investigate the effect of incorporating stinging nettle leaf flour at different levels (5%, 10%, 15%, and 20%) in the preparation of corn cookies. In addition, the storage stability of the formulated corn cookies was investigated at room (25 ± 2°C) and frozen (−18 ± 2°C) temperature. A control (100% corn cookies) was used to compare the nutritional, physical, and storage properties.

## 2. Materials and Methods

### 2.1. Raw Materials

Organic yellow corn (*Zea mays* L.) was obtained from the Institute of Agricultural Research for Development (IRAD), Yaounde (Cameroon). Stinging nettle leaves (*Urtica dioica* L.) were harvested on selected vegetable farms in Buea (Cameroon). Other ingredients, Jadida light margarine (Jadida, Med Oil, Cameroon), iodised salt (Saselsa, Cameroon), granulated sugar (SOSUCAM, Cameroon), semiskimmed powder milk (Nestlé NIDO, United Kingdom), 98% sodium bicarbonate (ALSA, Cameroon), and medium-sized eggs (grade 6), were purchased from local supermarkets. All chemicals and reagents used were of analytical grade.

### 2.2. Processing of Corn Seeds and Stinging Nettle Leaves to Flour

**Figure 1:**
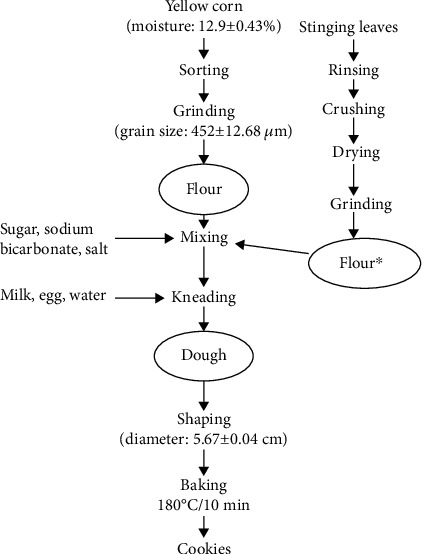
Formulation of corn and stinging leaves cookies. ∗For corn cookies incorporated with stinging nettle leaf flour, 5%, 10%, 15%, and 20% of the leaf flour were separately weighed and added during mixing.

Yellow corn and stinging nettle leaves were processed to flour as shown in Figure 1.

Organic yellow corn seeds (2 kg) were manually sorted for stones and splits. The seeds were then ground using an electric grinder (Atabong Ets, Cameroon) to obtain stone ground yellow corn flour (grain size: 452 ± 12.68 *μ*m).

Stinging nettle (*Urtica dioica* L.) was identified by its morphological characteristics, including bright, vibrant hairy green leaves with strongly serrated edges, and clearly visible venation [1]. The leaves were manually cut from the stems using a sterile pruning shear and placed in sterile polythene bags. Samples were transported to the laboratory in ice packs and processed within 1 h. Stinging nettle leaf flour was processed as described by Tanyitiku and Njombissie Petcheu [8]. Freshly harvested nettle leaves (25 g) were rinsed once in 225 mL of deionised water, drained, and crushed using an immersion blender (Breville, Australia; cutting speed, 30–40 mm/s; sharpening angle of the blade, 45°; and crushing time, 5–10 s). The crushed leaves were frozen at −18°C for 28 h and dried using a domestic freeze dryer (Harvest Right, United States) and set at −40°C for 30 h. The dry nettle leaves were then ground and sieved using an ASTM E11 400 *μ*m stainless steel to obtain stinging nettle leaf flour.

### 2.3. Cookie Preparation

Cookies were prepared according to AACC [14], as shown in Figure 1. Each formulation consisted of corn flour and constant ingredients in each formulation (margarine: 40 g, sugar: 30 g, milk: 20 g, salt: 1 g, 98% sodium bicarbonate: 1 g, deionised water: 20 mL, and a medium egg). Stinging nettle leaf flour was added at different levels to obtain four treatments as follows: 5NC (95 g corn flour + 5 g stinging nettle leaf flour), 10NC (90 g corn flour + 10 g stinging nettle leaf flour), 15NC (85 g corn flour + 15 g stinging nettle leaf flour), and 20NC (80 g corn flour + 20 g stinging nettle leaf flour). The control consisted of 100 g corn flour + 0 g stinging nettle leaf flour. The dry ingredients were mixed for 3 min using an electric mixer (Breville, Australia), rotating at 10,000 rpm, followed by the addition of wet ingredients until a dough was formed. Each mixture was rolled onto a cutting board and shaped into cookies (5.67 ± 0.04 cm in diameter) using a cookie cutter (size: 9.5 × 7.5 cm). The cookies were then evenly placed on baking trays, cooked in a preheated oven (Miele, Germany) at 180°C for 10 min, and allowed to cool to room temperature for 1 h. Subsequently, each formulated cookie was stored in labelled polyethene pouches (500 g per pouch) and stored at 4°C during analysis.

### 2.4. Proximate Composition of Stinging Nettle Leaves and Corn Cookies

Total ash, moisture, protein, and fat content was obtained according to AOAC [15]. Total ash was the remaining residue when 10 g of each sample was calcinated at 550°C for 24 h. Moisture was the difference in weight when 5 g of each sample was oven-dried at 105°C for 24 h. Crude protein was determined by the Kjeldahl method with a conversion factor of 6.25. Fat content was determined by the Soxhlet solvent extraction method using petroleum ether. For the starch content, digestible and resistant starch was measured using Megazyme digestible and resistant starch assay kit (Megazyme International, Ireland), according to manufacturer's guidelines.

### 2.5. Texture of Dough and Cookies

The texture of formulated dough and cookies was measured using a TA.XT Plus texture analyser (Stable Micro Systems, United Kingdom). It was equipped with a three-point bending rig (HDP/3 PB) with setting, 30 kg force in compression mode, 5 mm distance, 0.5 mm/s test speed, and 5 g of trigger force. After eight measurements per formulation, hardness (peak force) was determined as the maximum resistance of each dough or cookie to the rounded edge blade, and this occurred when the sample began to break [16].

### 2.6. Colour Profile

Cookie colour was measured using a HunterLab MiniScan EZ spectrophotometer (Hunter Associates Laboratory, United States), after five readings per formulation. Colour coordinates (*L*∗, *a*∗, and *b*∗) were interpreted as *L*∗ = lightness or darkness, *a*∗ = red to green (+*a* = redder, −*a* = greener), and *b*∗ = yellow to blue (+*b* = yellower, −*b* = bluer). Total colour difference, Δ*E*, between each sample and a blank (calibrated white tile) was obtained as follows.

 $$\begin{array}{c} \Delta E=\sqrt{{\left(\Delta L*\right)}^{2}+{\left(\Delta a*\right)}^{2}+{\left(\Delta b*\right)}^{2}} \end{array}$$

### 2.7. Spread Ratio and Baking Loss

The spread ratio was calculated by dividing the diameter of three sets of cookies by their thickness (height) [17]. In addition, the cookies were weighed immediately when shaped (*W*0) and then after cooking in the oven (*W*1). The percentage of baking loss was calculated as follows.

 $$\begin{array}{c} \text{Baking loss }\left(\%\right)=\frac{W0-W1}{W0}\times 100 \end{array}$$

### 2.8. Total Phenolic Content (TPC) and Antioxidant Activity (AA)

TPC and AA were measured for cookies before and after in vitro digestion (see Section 2.9) according to Tanyitiku and Njombissie Petcheu [8]. Both TPC and AA were measured before and after in vitro digestion, as recent studies have shown that polyphenols in particular could bind to starch or starch-digesting enzymes and reduce both in vitro and in vivo starch digestibility [18].

Sample extraction was carried out prior to each analysis. Each ground sample (2 g) was mixed with 10 mL of 80% ethanol. Under dark conditions, the mixture was incubated in a shaker incubator (Thermo Scientific, United States) at 25°C, 150 rpm for 24 h, and then centrifuged at 10,000 g for 10 min. The supernatant was collected, and the remaining sediment was mixed with another 10 mL of 80% ethanol and extracted again. The collected supernatants were combined and filtered using a Whatman No. 4 filter paper. Sample extracts were used for AA and phenolic assays.

TPC was measured by colorimetry using Folin–Ciocalteu (FC) reagent. Each sample extract (0.5 mL) was mixed with 5 mL of deionised water and 5 mL of FC phenol reagent for 3 min. In a shaker incubator, 2 mL of 10% Na_2_CO_3_ was added, stirred, and incubated at 30°C for 1 h. The absorbance at 765 nm was measured using a Hitachi spectrophotometer (Tokyo, Japan) with gallic acid (40–200 mg/L gallic acid) as standard. Results were expressed as milligram gallic acid equivalent (GAE) per gram of dry sample.

AA was estimated using a DPPH (2,2-diphenyl-1-picrylhydrazyl) free radical scavenging assay. Each sample extract (0.2 mL) was mixed with 2.5 mL of DPPH solution (0.35 mM DPPH dissolved in 50% ethanol) and incubated at room temperature for 10 min. The absorbance at 517 nm was measured, and the AA was calculated as a percentage inhibition with Trolox solution (100–1000 *μ*M) as standard. Results were expressed as micromole Trolox equivalents/gram of dry weight.

### 2.9. Estimated Glycaemic Index (GI) (eGI)

The eGI was determined as modified by Simons, Hall, and Tulbek [19]. Each sample (100 mg) was mixed with 5 mL of ethanol (80% *v*/*v*) in a test tube and incubated at 80 ± 3°C for 5 min. Another 5 mL of ethanol was added to the tube and centrifuged at 3000 rpm (1174 × g) for 10 min, and the supernatant was discarded. The sample was then resuspended in 10 mL of ethanol and again centrifuged at 3000 rpm (1174 × g) for 10 min. Subsequently, the residue was digested by adding *α*-amylase using the Megazyme resistant starch kit protocol (Megazyme International, Ireland). Each sample was placed in a shaker incubator, set at 37°C, and digested for 30, 60, 90, 120, 150, and 180 min. Reactions were terminated by adding 4 mL of 99% ethanol.

The amount of glucose released from starch at each interval was determined by spectrophotometry at 510 nm using a glucose oxidase-peroxidase (GOPOD) reagent from the Megazyme resistant starch kit. The hydrolysis index (HI) was calculated by dividing the area under the hydrolysis curve by the reference (white bread). The eGI was calculated according to [20] as follows.

 $$\begin{array}{c} \text{eGI}=39.71+0.549\text{HI} \end{array}$$

where eGI is the GI.

### 2.10. Storage Stability at Room and Frozen Temperature

Storage stability was evaluated by measuring water activity (*a*_*w*_), peroxide value (PV), and microbial count (aerobic plate count (APC), yeasts, and moulds) of stored cookie samples.

Each cookie formulation (500 g) was weighed and placed in separated airtight polyethylene pouches (volume: 1000 mL, thickness: 98 microns, and water vapour permeability: 1 g/m^2^). Based on Garden-Robinson [21], three separate pouches per formulation were stored for 6 months in two storage conditions: room temperature in a wooden cabinet and frozen at −18 ± 2°C (Haier, China). With each pouch sampled twice during the entire experiment, three samples per formulation were taken each month, thawed for 1 h (for frozen samples), and ground (12,000 rpm for 6 min) using a magic bullet mini blender (Nutribullet, United Kingdom). The blender was washed and cleaned in between samples to prevent cross-contamination.

Water activity was measured using a water activity meter (Aqualab, United States), according to manufacturer's guidelines. PV was measured according to AOAC [15] by titration. Each sample (5 g) was dissolved in 30 mL of glacial acetic chloroform solution (60:40 *v*/*v*) and incubated in a shaker incubator for 2 h. The solution was filtered with Whatman No. 1 filter paper. Subsequently, 30 mL of glacial acetic acid and 0.5 mL of saturated potassium iodide were added to 20 mL of the filtrate and allowed to stand for 30 min. Fifty milliliters of deionised water and 2–3 drops of 1% starch solution were then added and titrated with 0.01 N sodium thiosulfate with vigorous shaking until a colourless end point. The PV (milliequivalent per kilogram) was obtained as follows.

 $$\begin{array}{c} \text{Peroxide value}=\frac{\text{titre}\times \text{normality of sodium thiosulfate}}{\text{weight of sample}}\times 1000 \end{array}$$

APC, yeast, and moulds were enumerated using standard microbial culturing techniques [14]. Each sample (25 g) was homogenised in 225 mL of sterile buffered peptone water (BPW) using a stomacher. Subsequently, serial dilutions (10^−1^–10^−6^) using 1 mL of homogenate in 9 mL of BPW were prepared. For APC, samples were then cultured on plate count agar (Oxoid, United Kingdom) at 37°C for 48 h. Yeasts and moulds were enumerated by plating the dilutions on Potato Dextrose Rose Bengal Agar (Oxoid, United Kingdom) at 25°C for 5 days. Colonies were visually counted, and the results obtained were expressed in log_10_CFU/g.

### 2.11. Statistical Analysis

Unless specified, experiments were carried out in triplicate and analysed using IBM SPSS Statistics 23. Results were expressed as mean ± standard deviation, and mean differences were determined using ANOVA. When significant (*p* < 0.05) differences between means were observed, the means were separated using Tukey's test.

## 3. Results

### 3.1. Cookie Sample Composition

The proximate composition of the formulated corn cookies and stinging nettle leaf flour is presented in Table 1. The addition of stinging nettle leaf flour did not affect (*p* < 0.05) the moisture and resistant starch content of the formulated corn cookies. Ash and protein content increased from 0.32% (control) to 2.56% (20% stinging nettle incorporated) and 6.44% (control) to 21.52% (20% stinging nettle incorporated), respectively. On the other hand, fat content and digestible starch decreased from 2.13% (control) to 1.81% (20% stinging nettle leaf flour) and 31.89% (control) to 27.31% (20% stinging nettle incorporated), respectively.

### 3.2. Physical Characteristics of Corn Cookies

The control dough was harder (978.87 gf), and hardness significantly (p <0.05) decreased to 489.34 gf (20% nettle corn cookies) after stinging nettle flour incorporation (Table 2). Prior to baking, the hardness of 15% and 20% stinging nettle samples was significantly (*p* < 0.05) lower than the control corn cookies. After baking, the control maintained the highest hardness (1706.54 gf), while 15% enriched nettle became lowest (1326.63 gf). Baking loss could be correlated with cookie hardness as it was also highest for the control (8.98%) and varied between 6.93% and 8.23% for the nettle-enriched corn cookies. Similarly, the control corn cookies had a larger diameter (36.31 mm) and spread ratio (10.53), which significantly (*p* < 0.05) decreased with the incorporation of stinging nettle leaf flour.

The *L*∗, *a*∗, and *b*∗ values decreased with increasing incorporation of stinging nettle leaf flour, indicating higher lightness, redness, and yellowness for the control than for the nettle-enriched corn cookies. Lightness decreased from 54.93 (control) to 42.73 (20% nettle cookies) with 20% nettle-enriched corn cookies observed as the darkest cookies. Increasing stinging nettle leaf flour in corn cookies increased its greenness (−*a*∗ values) from a redder control corn cookie (*a*∗ = +0.21). Also, the control was the most yellow cookie (*b*∗ = 23.51), and the yellowness decreased significantly (*p* < 0.05) to *b*∗ = 23.51 (20% nettle) with an increase in substitution of nettle leaf flour. Further positive values for Δ*L*∗, Δ*a*∗, and Δ*b*∗ indicated that all formulated corn cookies were brighter, redder, and yellower, respectively, than the referenced calibrated white tile (*L*∗ = 81.79, *a*∗ = 1.08, and *b*∗ = 7.66). Δ*E* values significantly decreased with increasing substitution of nettle leaf flour and ranged between 6.11 (15% nettle) and 9.68 (control).

### 3.3. TPC and AA of Cookies

The AA and TPC values of stinging nettle-enriched corn cookies before and after digestion are presented in Table 3. Both AA and TPC values of all formulated corn cookies increased after in vitro digestion. Except for 5% nettle corn cookies, the TPC values were significantly (*p* < 0.05) different. In particular, TPC for 20% nettle corn cookies increased before digestion (120.32 mg GAE/g), and this further increase to about 27 times after digestion when compared with the control corn cookies.

### 3.4. Starch Hydrolysis and eGI

**Figure 2:**
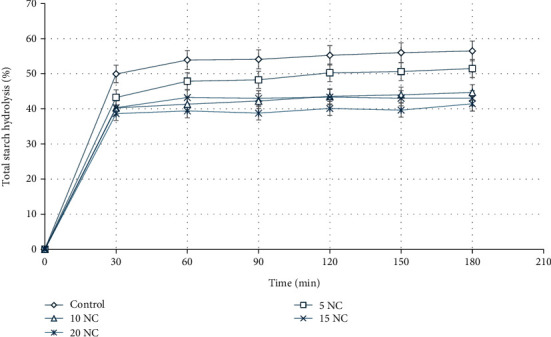
Starch hydrolysis of cookies during a 180-min in vitro digestion. Data are expressed as mean ± SD; control (100% corn), 5NC (95%corn + 5%nettle), 10NC (90%corn + 10%nettle), 15NC (85%corn + 15%nettle), and 20NC (80%corn + 20%nettle).

The starch hydrolysis of all formulated corn cookies could be divided into three phases—a sharp increase in hydrolysed starch during the first 30 min and a slight increase to 60 min which then flattened out up to 180 min of in vitro digestion (Figure 2). With this, all GI values were statistically (*p* < 0.05) different from each other, and the control was highest (eGI = 48.60) while 20% nettle corn cookies were lowest (eGI = 33.18) (Table 3). Specifically, the eGI values gradually reduced by 2%, 11%, 12%, and 15% with the increased incorporation of 5%, 10%, 15%, and 20% nettle leaf flour, respectively.

### 3.5. Storage Stability of Formulated Corn Cookies

**Figure 3:**
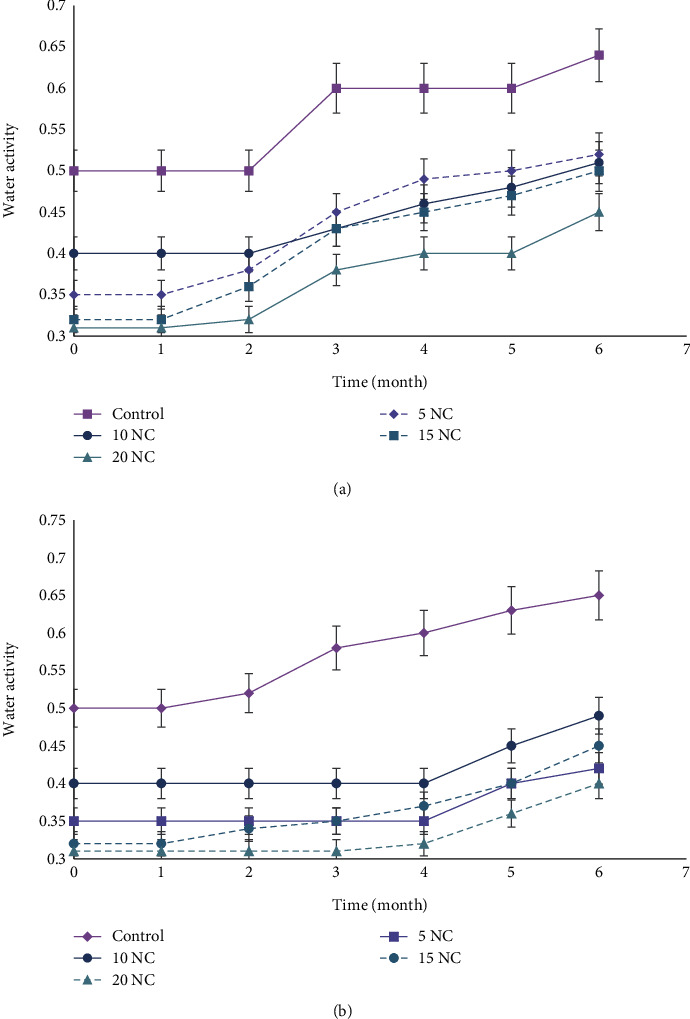
Water activity of corn cookies stored at room temperature and freezing degree: (a) at room temperature; (b) 18 ± 2°C. Data are expressed as mean ± SD; control (100% corn), 5NC (95%corn + 5%nettle), 10NC (90%corn + 10%nettle), 15NC (85%corn + 15%nettle), and 20NC (80%corn + 20%nettle).

The water activity (*a*_*w*_) of cookie samples is presented in Figure 3. On Day 0, the *a*_*w*_ of the formulated corn cookies was low and decreased (*p* < 0.05) after incorporation of nettle leaf flour. Control corn cookies had the highest *a*_*w*_ (0.51) and 20% nettle corn cookies the lowest (*a*_*w*_ = 0.31). During cookie storage, all *a*_*w*_ values were constant during the first month at both room and frozen storage conditions. At room temperature, the *a*_*w*_ of 5% and 15% nettle corn cookies then increased from the second month, while the control, 10%, and 20% corn cookies were not affected until the third month of *a*_*w*_ measurement. Between the second and third month, *a*_*w*_ significantly increase for all formulated cookies with 5% nettle corn cookies (initial *a*_*w*_ = 0.35) exceeding 10% nettle corn cookies (initial *a*_*w*_ = 0.40). For the frozen corn cookies, a similar increase in *a*_*w*_ was observed. However, except for the control and 15% nettle cookies that increased from the first month of storage, the *a*_*w*_ of 5%, 10%, and 20% nettle-enriched cookies remain constant up to the fourth month of storage.

**Figure 4:**
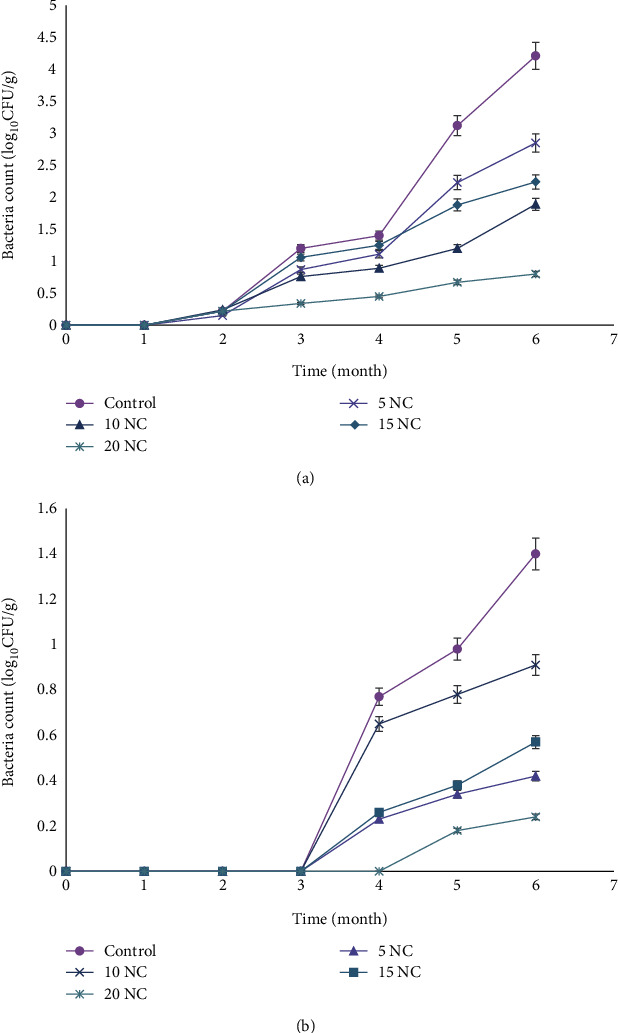
Bacterial count (log_10_CFU/g) of corn cookies stored at room temperature and freezing degree: (a) at room temperature; (b) 18 ± 2°C. Data are expressed as mean ± SD; control (100% corn), 5NC (95%corn + 5%nettle), 10NC (90%corn + 10%nettle), 15NC (85%corn + 15%nettle), and 20NC (80%corn + 20%nettle).

Furthermore, stored cookies were sampled every month and cultured for viable bacteria, yeasts, and moulds.  Figure 4 presents the mean APC of the formulated cookies during 6 months of storage at room and frozen temperature, respectively. Storage temperatures significantly affected bacterial growth, as more growth was observed at room temperature than at −18 ± 2°C. At room temperature, no bacterial growth was observed during the first month of storage. From the second month, a gradual increase in viable bacteria was observed, with control cookies having the highest growth (4.21 log_10_CFU/g) and 20% nettle-enriched corn cookies having the lowest (0.80 log_10_CFU/g) at the end of 6 months. For the frozen samples, bacterial growth was observed only after 3 months of storage and specifically at the fourth month for the 20% nettle-enriched cookies. Like at room temperature, the control was highest (1.4 log_10_CFU/g) while 20% nettle-enriched corn cookies were lowest (0.24 log_10_CFU/g) in total bacterial count.

**Figure 5:**
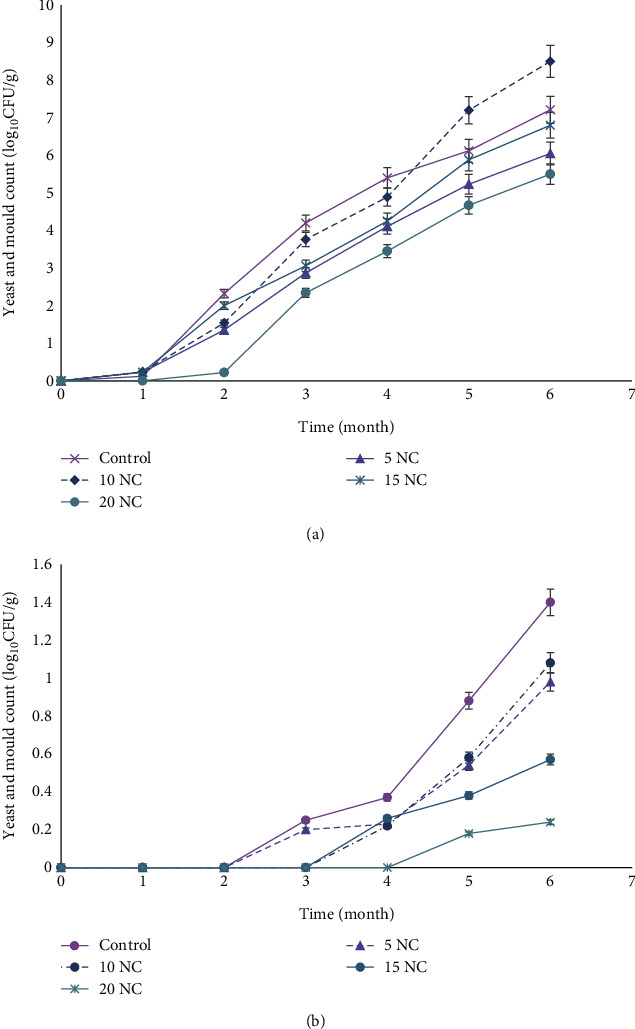
Yeast and mould count (log_10_CFU/g) of corn cookies stored at room temperature and freezing degree: (a) at room temperature; (b) 18 ± 2°C. Data are expressed as mean ± SD; control (100% corn), 5NC (95%corn + 5%nettle), 10NC (90%corn + 10%nettle), 15NC (85%corn + 15%nettle), and 20NC (80%corn + 20%nettle).

Like bacterial growth, a higher yeast and mould count was recorded at room temperatures than when frozen (Figure 5). Similarly, no growth was observed during the first month of storage at both temperatures. By the second month, growth was observed in all cookie samples stored at room temperature and 10% nettle corn cookies had the highest growth (8.50 log_10_ CFU/g) at the end of 6 months. Like water activity, 20% nettle corn cookies remained lowest when stored at room (5.50 log_10_ CFU/g) and frozen (0.24 log_10_ CFU/g) temperature.

**Figure 6:**
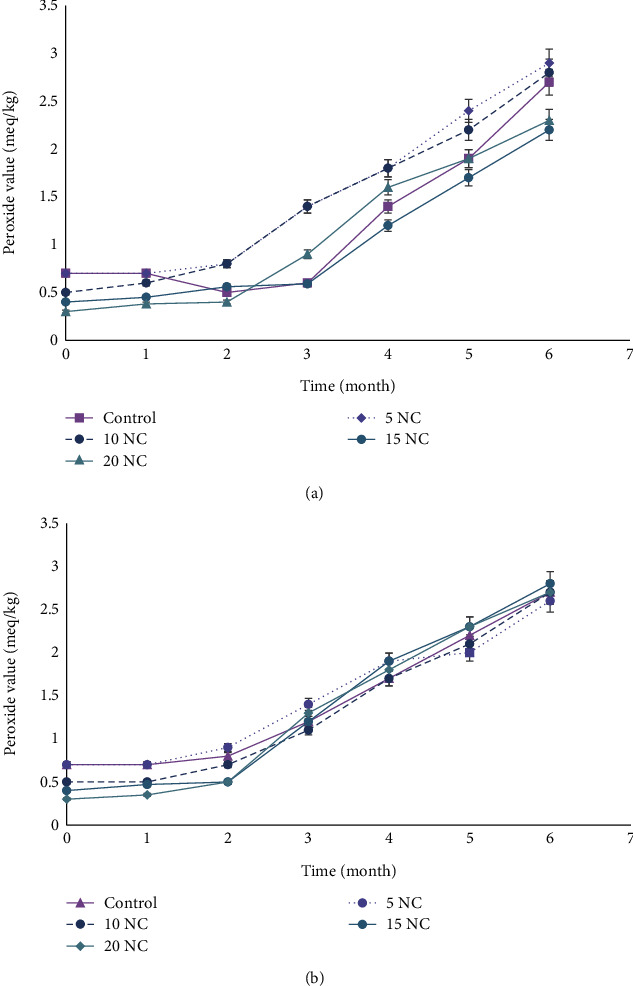
Peroxide value (meq/kg) of corn cookies stored at room temperature and freezing degree: (a) at room temperature; (b) 18 ± 2°C. Data are expressed as mean ± SD; control (100% corn), 5NC (95%corn + 5%nettle), 10NC (90%corn + 10%nettle), 15NC (85%corn + 15%nettle), and 20NC (80%corn + 20%nettle).

Also, the PVs increased with storage time and temperature (Figure 6). On Day 0, the PV of the control was highest (0.71 meq/kg) while 20% nettle was lowest (0.30 meq/kg). At room temperature, PV continued to increase till the end of 6 months with 5% and 10% nettle exceeding the control to 2.90 meq/kg and 2.81 meq/kg, respectively. Twenty percent nettle corn cookies remained the lowest at 2.22 meq/kg. On the other hand, the PV for the frozen cookie samples increased steadily until the end of 6 months with slight variations from each cookie sample.

## 4. Discussion

The incorporation of stinging nettle leaf flour improved the nutritional, physical, and shelf life characteristics of gluten-free corn cookies. In particular, the ash and protein contents were two and four times higher than the control, respectively. This increase could be due to the richer content of ash (11.23%) and protein (34.76%) of stinging nettle leaves. Man et al. [22] reported an increase in protein and ash when nettle leaf flour was incorporated into wheat bread. Likewise, Amadi [23] recorded an increase in protein (13.44%–17.03%) and ash (3.40%–4.84%) in wheat cookies enriched with moringa leaf flour. On the other hand, the results showed a decrease (*p* < 0.05) in digestible starch which could have been because of incorporating stinging nettle leaf flour of lower digestible starch (8.20%). Fat content also slightly decreased, and the effect was not (*p* < 0.05) significant. Maietti et al. [24] reported a similar decrease in fat (2.51–2.13 g/100 g) in wheat bread enriched with stinging nettles. In contrast, Hasrini, Aviana, and Khoiriyah [7] reported a significant increase in fat content (33.9 ± 0.93%–38.1 ± 1.2%) when herbal leaves such as kale, moringa, and katuk were incorporated into modified cassava flour cookies (mocaf).

Stinging nettle-enriched corn cookies appeared to produce softer cookies (less hardness) than the control. Evdokimova et al. [25] reported an increase in viscosity index of 25.3%, 39.2%, and 42.6% in wheat dough when nettle flour was incorporated at 1%, 2%, and 3%, respectively. As such, a softer cookie in this research indicated that the viscosity of the nettle-enriched corn dough could have remained stable during cooking. Sahagún and Gómez [26] reported similar cookie hardness when peas, potatoes, and whey protein were incorporated into corn flour cookies (8.41% moisture, 4.58% protein). Likewise, Tarasevičienė et al. [27] reported increased softness after the addition of raspberry, red currants, and strawberry pomace flour in wheat cookies. In contrast, Swapnil et al. [28] reported an increase in cookie hardness (from 4.567 to 6.432 N) after the incorporation of moringa leaf powder. Moreover, this was correlated to the decrease in gluten that will usually form viscoelastic networks during the hydration of wheat flour [28].

During baking, baking loss constitutes the lost in water and organic material [16]. Changes in cookie dimension are observed due to the spreadability of the flour, the action of leavening agent, and the influence of heat and sugars dissolved [17]. In the same process, a change in cookie colour could occur due to Maillard reactions between reducing sugars and the amino side chain of lysine [26, 27] and during the oxidation and caramelization of nettle pigments and phenolics [29]. Baking loss and cookie spread were highest in the control and decreased (*p* < 0.05) with stinging nettle leaf flour incorporation. Wójcik et al. [13] and Man et al. [22] also reported a decrease in wheat bread volume with the incorporation of nettle leaf flour. This was attributed to an increase in dilution of wheat gluten content, as well as the interactions between the fibre, water, and gluten components of the formulated bread [22]. Like in this research, Stamatovska et al. [30] recorded very visible colour changes (Δ*E* > 6) in biscuits made from 70% barley flour (Δ*E* = 10.89), 100% barley flour (Δ*E* = 9.09), and 100% wheat flour (Δ*E* = 7.47) after 5–7 min of baking. A similar decrease in *L*∗, *a*∗, and *b*∗ values was reported in sponge cake enriched with nettle leaf powder [29]. The variation in cookie colour in this study strongly indicated that the *L*∗, *a*∗, and *b*∗ values were greatly influenced by beta carotene in yellow corn seeds [1] and the green chlorophyll pigment in nettles [31].

Furthermore, significant (*p* < 0.05) increase in TPC and AA values was observed before and after in vitro digestion. Maietti et al. [24] reported a similar increase in TPC and AA values from 372 *μ*gGAE/g (white bread) to 597 *μ*gGAE/g and 0.53 mgTE/g (white bread) to 0.83 mgTE/g, respectively, in nettle-enriched wheat bread. Likewise, Mitrović et al. [32] recorded a significantly (*p* < 0.05) higher free phenolic content (2537.18 *μ*g/g) in wheat cookies enriched with nettle seed, and this was 21.5% higher than the phenolic content of the control wheat cookies (1991.75 *μ*g/g). Polyphenols and antioxidants exert beneficial effects on health conditions such as cancer, diabetes, and cardiovascular disease by retarding carbohydrate digestion and glucose absorption in the small intestine [24, 33]. Adhikari, Bajracharya, and Shrestha [34] demonstrated that nettle leaf powder contained higher TPC (129 mgGAE/g) and AA (66.3 DPPH % inhibition) than wheat (1.3 mgGAE/g and 23.72 DPPH % inhibition) and barley (1.7 mgGAE/g and 28.64 DPPH% inhibition), respectively. As such, the findings strongly indicated that the consumption of nettle-enriched gluten-free corn cookies could provide significantly higher levels of polyphenols and antioxidants, leading to its associated health benefits in humans.

The digestible starch and rate of starch hydrolysis of the control cookie samples were higher than that of all corn cookies enriched with nettle. This could be because the substitution of corn flour with stinging nettle leaf flour reduced the amount of hydrolysed starch in the final nettle-enriched corn cookies. Also, the action of nettle fibres with gelatinized starch could have led to the formation of a polymeric matrix that made the attack of *α*-amylase more difficult during in vitro digestion [20]. Correspondingly, significant (*p* < 0.05) lower expected GI values were obtained for the formulated nettle-enriched corn cookies. Leoro Vernaza et al. [20] reported a reduction in GI of up to 50% when passion fruit fibre was gradually incorporated into extruded corn breakfast cereals. Likewise, Triandita and Putri [35] reported a 40% decrease in GI when black soybean flour was incorporated into corn cookies. The expected GI indicates how quickly the blood glucose level is affected after the consumption of starchy foods. Foods with GI greater than 71 are found to be closely associated with degenerative diseases such as Type 2 diabetes, cardiovascular disease, and obesity [35, 36]. Corn is a starchy food, and 100% corn cookies are not an exception to having a higher GI due to their starch content (Table 1). However, lower eGI values in this research indicated that nettle-enriched corn cookies could be considered as “low GI products” with potential health benefits to diabetic patients in controlling postprandial insulin release [20, 35].

Importantly, to estimate the shelf life of the formulated corn cookies, water activity, PV, total viable bacteria, yeast, and moulds during 6 months of cookie storage were investigated. Cookie shelf life was considered as the duration in which formulated corn cookies could be safely consumed when stored at room or frozen temperature [21]. The results of water activity were consistent with those of Ruszkowska, Tańska, and Kowalczewski [37], who obtained water activity ranging between 0.311 and 0.386 when cricket powder was incorporated into extruded corn snacks. In contrast, Mitrevski et al. [38] reported a decrease in water activity from 0.501 to 0.347 when 20% beetroot-enriched spelt flour biscuits were stored for 3 months. Water activity is a measure of the water available for biological reactions and indicates the shelf life of food products. For example, food spoilage bacteria will normally grow when *a*_*w*_ is greater than 0.91, while moulds and yeasts will grow when *a*_*w*_ is less than 0.80 [38].

Microbial spoilage is very critical in limiting the texture, colour, flavour, and shelf life of food. In fact, the maximum microbial limit in baked products (cake, bread, cookies, and biscuits) is < 10^5^ CFU/g for total plate count and < 104 CFU/g for yeast and moulds [39]. To maintain the quality and shelf life of cookies, Garden-Robinson [21] recommends a storage of 3 weeks at room temperature or 6 months when frozen. In this research, no microbial growth was observed within the first month of storage under both conditions, indicating cookies stored at room temperature were within acceptable consumption limits at the end of 1 month. Subsequently, the minimal onset of microbial growth at the second and third months of frozen cookie storage also indicated that the formulated corn cookies were within acceptable limits for human consumption [39].

In terms of PV, Asadi, Khan, and Zaidi [40] reported a similar increase in PV (1.04–4.98 meq/kg) when beetroot leaf powders were incorporated into wheat cookies and stored for 15 months. This was attributed to an increase in oxygen migration into the packaging, which resulted in fat oxidation and slight rancidity [40]. Similarly, Kaur, Choudhary, and Sharma [41] reported an increase from 0.65 and 0.50 meq/kg to 3.60 and 3.74 meq/kg in sweet wheat cookies when stored for 90 days under packaging and unpackaging conditions, respectively. Notwithstanding, the PVs of all formulated corn cookies stored at both room and frozen temperature were within the recommended permissible limits of < 10 meq/kg [41].

Although further research is required to understand the rheological, viscosity, and structural properties of stinging nettle leaf flour incorporated corn cookies, a low water activity, microbial count, and PVs indicated that this research formulated gluten-free corn cookies could maintain their quality and shelf life characteristics within the recommended room and freezing storage conditions.

## 5. Conclusions

Gluten-free corn cookies of improved nutritional, physical, and storage characteristics were developed. Specifically, the substitution of corn flour with stinging nettle leaf flour produced cookies with approximately 15 times lower expected GI than 100% corn cookies. Although further research is required to understand the functional, structural, and sensory properties of the formulated gluten-free corn cookies, these research findings strongly indicated the nutritional and potential health benefits of stinging nettle leaves, as a food ingredient in gluten-free bakery products.

## Figures and Tables

**Table 1:** Macronutrients in stinging nettle leaf flour and formulated cookies.

**Composition**	**SNLF**	**Control**	**5NC**	**10NC**	**15NC**	**20NC**
Ash (%)	11.23 ± 0.04^a^	0.32 ± 0.46^e^	1.89 ± 0.55^c^	2.05 ± 0.31^cd^	2.33 ± 0.04^cd^	2.56 ± 0.60^b^
Moisture (%)	6.51 ± 0.76^b^	9.92 ± 0.19^ac^	8.79 ± 0.63^a^	8.64 ± 0.88^a^	8.71 ± 1.08^a^	9.70 ± 1.21^ac^
Protein (%)	34.76 ± 1.22^a^	6.44 ± 0.93^e^	18.58 ± 0.27^d^	20.65 ± 0.43^c^	20.05 ± 0.9^c^	21.52 ± 0.04^b^
Fats (%)	0.33 ± 0.08^c^	2.13 ± 0.52^a^	2.01 ± 0.14^a^	2.10 ± 0.01^a^	1.94 ± 0.33^b^	1.81 ± 0.12^b^
Digestible starch (%)	8.20 ± 0.45^e^	31.89 ± 0.17^a^	30.61 ± 0.32^b^	26.83 ± 0.24^d^	28.69 ± 0.04^c^	27.31 ± 0.27^c^
Resistant starch (%)	5.45 ± 1.34^a^	2.91 ± 0.70^b^	2.42 ± 0.02^b^	2.23 ± 0.31^b^	2.97 ± 0.52^b^	3.78 ± 0.44^b^

*Note:* Values are mean ± standard deviation of three replications. Means with different superscripts within the same row are significantly (*p* < 0.05) different.

Abbreviations: SNLF: stinging nettle leaf flour; control: 100% corn; 5NC: 95%corn + 5%SNLF; 10NC: 90%corn + 10%SNLF; 15NC: 85%corn + 15%SNLF; 20NC: 80%corn + 20%SNLF.

**Table 2:** Baking loss, spread ratio, hardness, and colour of cookies.

**Parameter**	**Control**	**5NC**	**10NC**	**15NC**	**20NC**
Baking loss (%)	8.98 ± 0.04^a^	7.33 ± 0.01^c^	8.23 ± 0.04^b^	7.18 ± 0.00^d^	6.93 ± 0.02^e^
Diameter (mm)	36.31 ± 0.41^a^	34.23 ± 0.26^b^	30.21 ± 0.12^e^	33.89 ± 0.52^c^	31.83 ± 0.55^d^
Thickness (mm)	3.45 ± 0.02^a^	3.41 ± 0.81^a^	3.57 ± 0.31^a^	3.59 ± 0.22^a^	3.48 ± 0.08^a^
Spread ratio	10.53 ± 0.01^a^	10.04 ± 0.08^b^	8.46 ± 0.11^e^	9.44 ± 0.02^c^	9.16 ± 0.12^d^
Hardness (gf)					
Dough	978.87 ± 39.60^a^	965 ± 88.23^a^	696.42 ± 61.90^b^	557.71 ± 35.87^c^	489.34 ± 62.15^c^
Cookies	1706.54 ± 445.15^a^	1543.76 ± 321.61^a^	1463.34 ± 704.10^ac^	1326.63 ± 925.31^ab^	1491.09 ± 152.44^ac^
Colour					
*L*∗	54.93 ± 0.61^a^	50.10 ± 0.38^b^	45.31 ± 0.44^c^	44.02 ± 0.53^c^	42.73 ± 0.32^d^
*a*∗	0.21 ± 0.36^d^	−0.65 ± 0.18^b^	−0.24 ± 0.11^c^	−0.88 ± 0.72^b^	−1.40 ± 0.27^a^
*b*∗	23.51 ± 0.20^a^	20.51 ± 0.25^b^	15.43 ± 0.08^c^	10.46 ± 0.04^d^	8.74 ± 0.32^e^
Δ*E*	9.68 ± 0.42^a^	8.09 ± 0.04^b^	6.21 ± 0.34^c^	6.11 ± 0.52^c^	5.86 ± 0.17^d^

*Note:* Values are mean ± standard deviation of three replications; means with different superscripts within the same row are significantly (*p* < 0.05) different.

Abbreviations: Control: 100% corn; 5NC: 95%corn + 5%nettle; 10NC: 90%corn + 10%nettle; 15NC: 85%corn + 15%nettle; 20NC: 80%corn + 20%nettle.

**Table 3:** TPC, DPPH, and estimated glycaemic index (eGI) of corn cookies.

**Formulation**	**TPC (mg GAE/g)**		**DPPH (*μ*mol TE/g)**		**eGI**
	**Before digestion**	**After digestion**	**Before digestion**	**After digestion**	
Control	89.22 ± 0.08^d^	102.55 ± 0.01^e^	4.32 ± 0.08^e^	6.42 ± 0.02^e^	48.60 ± 0.02^a^
5NC	84.95 ± 5.66^e^	112.52 ± 6.45^d^	5.30 ± 0.54^d^	8.40 ± 0.64^d^	45.81 ± 0.08^b^
10NC	102.33 ± 2.98^c^	120.28 ± 0.34^c^	8.10 ± 0.02^c^	9.8 ± 0.31^c^	37.98 ± 0.04^d^
15NC	118.02 ± 1.38^b^	125.32 ± 2.17^b^	9.50 ± 0.21^b^	10.93 ± 0.45^b^	38.72 ± 0.00^c^
20NC	120.32 ± 0.08^a^	129.88 ± 0.04^a^	10.99 ± 0.38^a^	14.22 ± 0.33^a^	33.18 ± 0.02^e^

*Note:* Data are expressed as mean ± SD; means with different superscripts within the same column are significantly (*p* < 0.05) different.

Abbreviations: Control: 100% corn; 5NC: 95%corn + 5%nettle; 10NC: 90%corn + 10%nettle; 15NC: 85%corn + 15%nettle; 20NC: 80%corn + 20%nettle.

## Data Availability

The data that support the findings of this study are available from the corresponding author upon reasonable request.
